# Waiting for baseline stability in single-case designs: Is it worth the time and effort?

**DOI:** 10.3758/s13428-022-01858-9

**Published:** 2022-04-25

**Authors:** Marc J. Lanovaz, Rachel Primiani

**Affiliations:** 1 École de psychoéducation, Université de Montréal, C.P. 6128, succursale Centre-Ville, Montreal, QC, H3C 3J7 Canada; 2 Centre de recherche de l’Institut universitaire en santé mentale de Montréal, Montreal, Canada

**Keywords:** AB design, Baseline, Data analysis, Machine learning, n-of-1 trial, Single-case design

## Abstract

Researchers and practitioners often use single-case designs (SCDs), or n-of-1 trials, to develop and validate novel treatments. Standards and guidelines have been published to provide guidance as to how to implement SCDs, but many of their recommendations are not derived from the research literature. For example, one of these recommendations suggests that researchers and practitioners should wait for baseline stability prior to introducing an independent variable. However, this recommendation is not strongly supported by empirical evidence. To address this issue, we used Monte Carlo simulations to generate graphs with fixed, response-guided, and random baseline lengths while manipulating trend and variability. Then, our analyses compared the type I error rate and power produced by two methods of analysis: the conservative dual-criteria method (a structured visual aid) and a support vector classifier (a model derived from machine learning). The conservative dual-criteria method produced fewer errors when using response-guided decision-making (i.e., waiting for stability) and random baseline lengths. In contrast, waiting for stability did not reduce decision-making errors with the support vector classifier. Our findings question the necessity of waiting for baseline stability when using SCDs with machine learning, but the study must be replicated with other designs and graph parameters that change over time to support our results.

Researchers in the healthcare and behavioral sciences are increasingly using single-case designs (SCD) to develop and validate novel treatments (McDonald & Nikles, 2021; Rader et al., 2021). When testing the effects of a treatment or intervention, SCDs aim to demonstrate the presence of a functional relation between the introduction of a treatment and a change in behavior or other relevant outcomes (Horner et al., 2005; Tate et al., 2013). At this point, the reader should note that that the utility of these designs is not limited to researchers: Practitioners may also use SCDs to monitor the effects of treatment or intervention (Mitteer et al., 2018). In SCDs, an experimenter exposes one or more participants to two or more conditions. The first condition (phase A), which is comparable to the control group in a group design, consists of repeatedly measuring the dependent variable (e.g., behavior, product) prior to introduction of treatment. On the other hand, phase B involves the implementation of the treatment while continuing the repeated measurement of the dependent variable (Ledford & Gast, 2018). This sequence, referred to as an AB comparison, is particularly important in SCDs because it represent the basic unit of multiple experimental designs. For example, the AB comparison is central to the demonstration of functional relations in reversal (ABAB) designs, in multiple baseline designs, and in changing-criterion designs.

When analyzing the repeated changes between phases A and B, researchers and practitioners may identify the presence of a functional relation if the independent variable generates reliable and consistent changes in the dependent variable (Fisher et al., 2003; Vannest et al., 2018). When assessing the effects of a treatment, the demonstration of a functional relation indicates that it is at least partly effective. On the contrary, the lack of a functional relation indicates that the treatment may be ineffective. To conduct these analyses, many researchers and practitioners use visual inspection despite its limitations and sometimes poor accuracy (Falligant et al., 2020; Lanovaz & Hranchuk, 2021; Ninci et al., 2015). Visual raters rely on multiple data features to help them identify the presence of a functional relation such as comparing level, trend, variability, consistency and overlap across phases (Kratochwill et al., 2010; Manolov & Vannest, 2019). A functional relation is said to exist when one or more of these characteristics change consistently across replications (Ledford et al., 2019).

When implementing SCDs, researchers and practitioners typically follow guidelines to design their procedures. For example, *What Works Clearinghouse* (WWC; 2020) developed the most highly cited guidelines (Kratochwill et al., 2010), which provide guidance on how to implement SCDs. When using SCDs, an issue that researchers and practitioners must deal with involves trend stability during baseline. That is, too much trend during baseline may obscure changes observed in subsequent phases. One solution proposed by the original WWC guidelines involves “waiting to see whether the [baseline] series stabilizes as more data are gathered” (Kratochwill et al., 2010, p.19–20). This manipulation facilitates the comparison of patterns across phases, especially when using visual inspection. Some of the most popular introductory textbooks for teaching single-case designs (e.g., Barlow et al., 2009; Cooper et al., 2020; Kazdin, 2011; Ledford & Gast, 2018) also recommend waiting for baseline stability prior to introducing treatment.

Although this “response-guided” approach is commonly recommended, waiting for stability may lead to reductions in variance, which may have an impact on the analyses (Swan et al., 2020). Furthermore, having an experimenter selecting the “right” time to introduce a baseline may actually increase type I error rates when using visual inspection and randomization tests (Allison et al., 1992; Byun et al., 2017; Ferron et al., 2003; Todman & Dugard, 1999). In other words, waiting for stability may increase the probability of concluding that a graph shows a change when no true change has occurred. Nevertheless, the extent to which response-guided decisions in baseline increase type I error rate remains to be further validated with analyses methods beyond randomization tests and visual inspection.

Recently, researchers have examined the use of a blind response-guided approach to analyze multiple baseline graphs, which was referred to as masked visual analysis (Ferron et al., 2017). Masked visual analysis involves randomly introducing the independent variable within a random tier of a multiple baseline design when data show stability in all tiers, and subsequently introducing it in other tiers when the visual raters conclude that the treatment was introduced in a tier. Then, the probability of the blind raters specifying the correct treatment order at random can be computed. If this value is less than .05 and the blind raters identify the correct treatment order, researchers may deem that the graphs as showing a clear functional relation. Promisingly, the masked visual analysis can be applied to a variety of designs such as multiple baseline, reversal, and changing-criterion designs (Byun et al., 2017; Fallon et al., 2020; Ferron et al., 2017; Ferron et al., 2019; Joo et al., 2018). That said, the main limitation of masked visual analysis is that it continues to rely on visual inspection, which does not always produce consistent results and is difficult to apply on very large datasets (Fisher et al., 2003; Lanovaz & Hranchuk, 2021; Wolfe et al., 2016).

Our study examined the effects of response-guided decision-making on the most basic unit of analysis, the AB comparison. Given that the multiple baseline, reversal, and changing-criterion designs are based on the repetition of this unit, studying how waiting for baseline stability affects decision-making errors in AB designs may produce results that are generalizable to multiple experimental designs. As indicated earlier, prior studies on the topic have limited their analysis to randomization tests and visual inspection. However, multiple other methods exist to analyze single-case graphs. To extend prior research, we selected two methods that have never been used to examine response-guided decision-making for single-case designs: the conservative dual-criteria (CDC; Fisher et al., 2003) and machine learning (Hranchuk & Lanovaz., 2021). Both these methods perform well with short data series, which may make them relevant to examine the effects of response-guided decisions. Analysis methods may behave differently on graphs with various characteristics (e.g., trend vs. no trend), which is why testing more than one method appears important (Manolov & Vannest, 2019). In sum, our study compared the type I error rate and power of two methods of analyses for AB graphs with fixed baseline lengths, response-guided baseline lengths, and random baseline lengths for sets of graphs with different characteristics.

## Experiment 1 – Waiting for stability in trend

One type of stability in single-case designs involves trend. When using single-case designs, researchers and practitioners typically wait for baseline to show minimal-to-no trend before introducing the independent variable (Ledford & Gast, 2018). The purpose of the first experiment was to examine the effects of waiting for baseline trend stability in datasets that showed an initial trend resulting from random variations.

### Method

The first step of the method involved generating time series and AB graphs by conducting Monte Carlo simulations. Next, we applied the CDC method and the support vector classifier (i.e., a machine learning algorithm) to determine whether each graph showed an effect or not. Finally, our analyses compared type I error rates and power across graphs with fixed, response-guided, and random baseline lengths. All our code is freely available under a MIT permissive software license: 10.17605/OSF.IO/H7BSG.

#### Monte Carlo simulation

Our study used a Monte Carlo approach to simulate a total of 160,000 time series that contained 30 points each. To generate our time series, we programmed Python (version 3.7.7) to use the following formula:

$${\displaystyle \begin{array}{c}{y}_t={x}_t+10\\ {}\mathrm{where}\ {x}_t=a{x}_{t-1}+{\varepsilon}_t\end{array}}$$

In the formula, *x*_*t*_ represents the autocorrelated value at time point *t*, which is composed of (a) the autocorrelated value from the previous time point, *x*_*t* – 1_, (b) a lag 1 autocorrelation of *a,* and (c) an error term, ε_*t*_, randomly generated from a normal distribution with a mean of 0 and standard deviation of 1. To produce our final values, *y*_*t*_, the code added a constant of 10 to prevent graphs with negative values. Half the time series contained no autocorrelation whereas the other half had an autocorrelation value of 0.4, which is the approximate mean autocorrelation reported in a recent study on single-case graphs (Barnard-Brak et al., 2021a). We focused on first-order autocorrelations as more proximal events tend to produce larger effects than more distal events when analyzing behavior (Cooper et al., 2020). Each initial time series contained exactly 30 points.

As the purpose of our study was to examine whether waiting for stability produced more or fewer errors than simply implementing the treatment when the trend was still unstable, our analyses required initially unstable time series. During data generation, we only kept time series that showed a trend above a maximum absolute threshold after a specific minimum number of points in baseline. The maximum allowable absolute threshold was set at 15 degrees for half the time series and at 30 degrees for the other half. The minimum number of points for phase A was equally distributed with values of either 3 or 5. We selected these values because applied researchers and practitioners typically want to reduce their number of baseline sessions to minimize the inconvenience to their clients (Lanovaz et al., 2017). The code involved a loop that continued creating novel 30-point series that met the previous criteria. As an example, assume that the maximum allowable trend was 30 degrees and the series had to have a minimum of five points in baseline. In this case, we would only keep a time series if it showed a trend larger than 30 degrees, or smaller than – 30 degrees, after five points.

The next step involved transforming these time series to AB graphs. Each 30-point data series yielded three separate AB graphs. The first graph involved setting the number points in phase A at the minimum (i.e., three or five), which yielded a graph with a fixed baseline length. Then, we added either 5 or 10 points in phase B to the graph because treatment phases tend to be longer than baseline phases. Therefore, the AB graph did not use all the points from the times series. For example, a graph with three points in phase A and ten points in phase B would only use the first 13 points in the time series^1^ for the fixed baseline length. For half the series, we added a standardized mean difference (SMD) value to phase B, which simulated an effect to test for power. This SMD value was uniformly distributed integers from 1 to 5, inclusively. For the other half of the series, we did not simulate an effect (SMD = 0) to examine type I error rate.

The second graph required waiting for stability using the same time series (i.e., response-guided decision to terminate phase A). That is, phase A ended when the trend was below the absolute value of the maximum allowable trend. Thus, the response-guided graphs always contained more points in phase A than the fixed baseline graphs. The other values (i.e., number of points in phase B and SMD) remained the same as for the first graph designed using the same time series. Using the same time series and keeping the other values constant allowed us to control the effects of waiting for stability. If phase A achieved stability after seven points and the phase B was set at ten points, the response-guided AB graph would contain 17 points (i.e., the first 17 points of the same 30-point series).

The third graph consisted of randomly selecting the number of points in phase A. To control for the potential confound of phase lengths, the frequency distribution of these phase lengths were exactly the same as the one for the response-guided graphs. That is, we picked the phase lengths randomly without replacement from the distribution of phase lengths observed in the responded-guided baselines. The SMD and number of points in phase B remained the same as for the first and second graphs derived from the same time series.

Our code repeated this process for 160,000 times series creating an initial dataset containing a total of 480,000 AB graphs. Table 1 presents the characteristic distributions of these times series. Note that the characteristics of the time series graphs were perfectly counterbalanced to control for interaction effects. Our dataset contained 5000 time series for each combination of characteristics showing no effect and 5000 times series for each set of characteristics simulating an effect (i.e., 1000 for each of the five SMD values).

**Table 1:** Characteristics manipulated across times series

Characteristic	Values	
Minimum number of points in phase A (fixed)	Shorter: 3 points (*n* = 80,000) Longer: 5 points (*n* = 80,000)	
Number of points in phase B	Shorter: 5 points (*n* = 80,000) Longer: 10 points (*n* = 80,000)	
Autocorrelation	Autocorrelation absent: *a* = 0.0 (*n* = 80,000) Autocorrelation present: *a* = 0.4 (*n* = 80,000)	
Maximum allowable trend	Small: 15 degree (*n* = 80,000) Large: 30 degrees (*n* = 80,000)	
Effect size	No effect: With effect:	SMD = 0 (*n* = 80,000) SMD = 1 (*n* = 16,000) SMD = 2 (*n* = 16,000) SMD = 3 (*n* = 16,000) SMD = 4 (*n* = 16,000) SMD = 5 (*n* = 16,000)

*Note. a*: autocorrelation coefficient, *n*: number of times series with the value, SMD: standardized mean difference.

#### Analyses

To identify whether each graph showed a clear change, we applied the two objective methods that have been shown to perform best on baselines with few data points: the CDC method (Fisher et al., 2003) and a support vector classifier developed by Lanovaz and Hranchuk (2021). The CDC relies heavily on level and trend whereas the support vector classifier considers additional dimensions of data analysis such as variability. The CDC method involved tracing a continuation of mean and trend lines from phase A unto phase B and increasing their height by 0.25 standard deviations. Then, we counted the number of points that fell above both lines. If this number of points was equal or higher than a threshold based on the binomial distribution, the graph was labeled as showing an effect. Otherwise, the graph was categorized as showing no effect.

The support vector classifier is a machine learning algorithm that involves projecting the data into a higher dimension and then separating them with a hyperplane (i.e., a plane projected in more than two dimensions). The current study did not train the classifier: we simply downloaded the model previously developed by Lanovaz and Hranchuk (2021) and applied it to our data. Prior to its application, our code transformed the graphs to *z* scores and extracted the eight features required by the classifier: means of phases A and B, standard deviations of phases A and B, intercept and slope of phase A, and intercept and slope of phase B. Based on this input, the classifier categorized each graph as either showing an effect (1) or showing no effect (0).

Our initial analysis involved examining type I error rate and power across graphs having the minimum number of points (i.e., fixed graphs), response-guided graphs, and graphs having a random number of points in phase A. Computing type I error involved dividing the number of graphs with a SMD of 0 that showed an effect according to the method of analysis (i.e., CDC or support vector classifier) by the total number of graphs with a SMD of 0. This measure represents the probability of concluding that a graph shows an effect when the graph includes no true effect (false positives). To compute power, we instructed Python to divide the number of graphs with a SMD of 1 or more that showed an effect according to the method of analysis by the total number of graphs with a SMD of 1 or more. Power identifies the proportion of graphs with a true effect that were correctly categorized as such by each method of analysis.

Following our main analysis, we examined the effects of initial confounding trend (i.e., decreasing or increasing) on type I error rate and power. This analysis assumed that the initial trend (i.e., trend following the minimum number of points) could differentially affect each method. The final analysis involved examining the impact of each characteristic manipulated during time series generation: minimum number of points in phase A, number of points in phase B, autocorrelation, maximum absolute allowable trend, and standardized mean differences. 

### Results and discussion

**Fig. 1:**
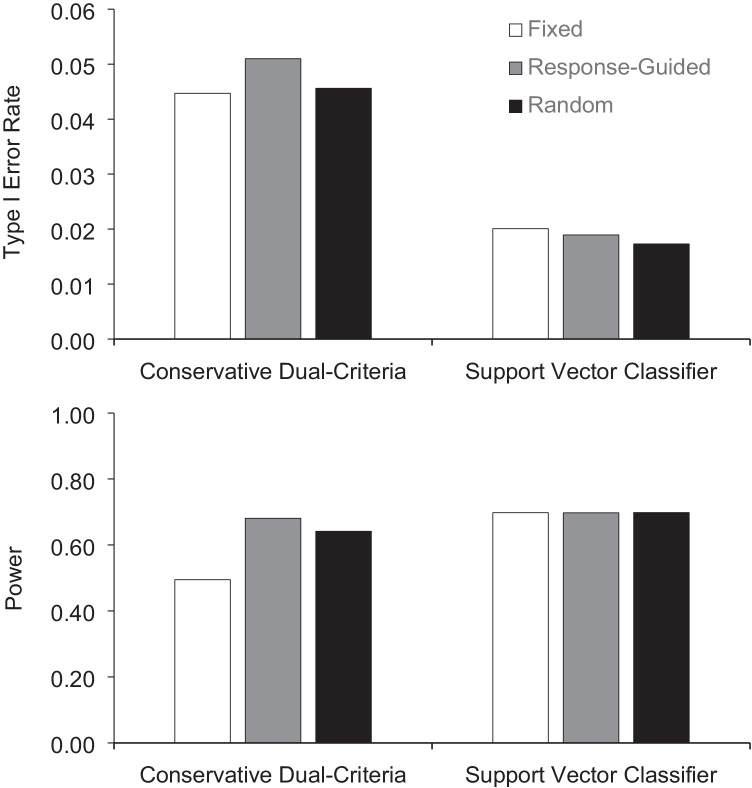
Type I error rate and power across fixed, response-guided, and random baseline lengths for the conservative dual-criteria and support vector classifier for trend stability

**Fig. 2:**
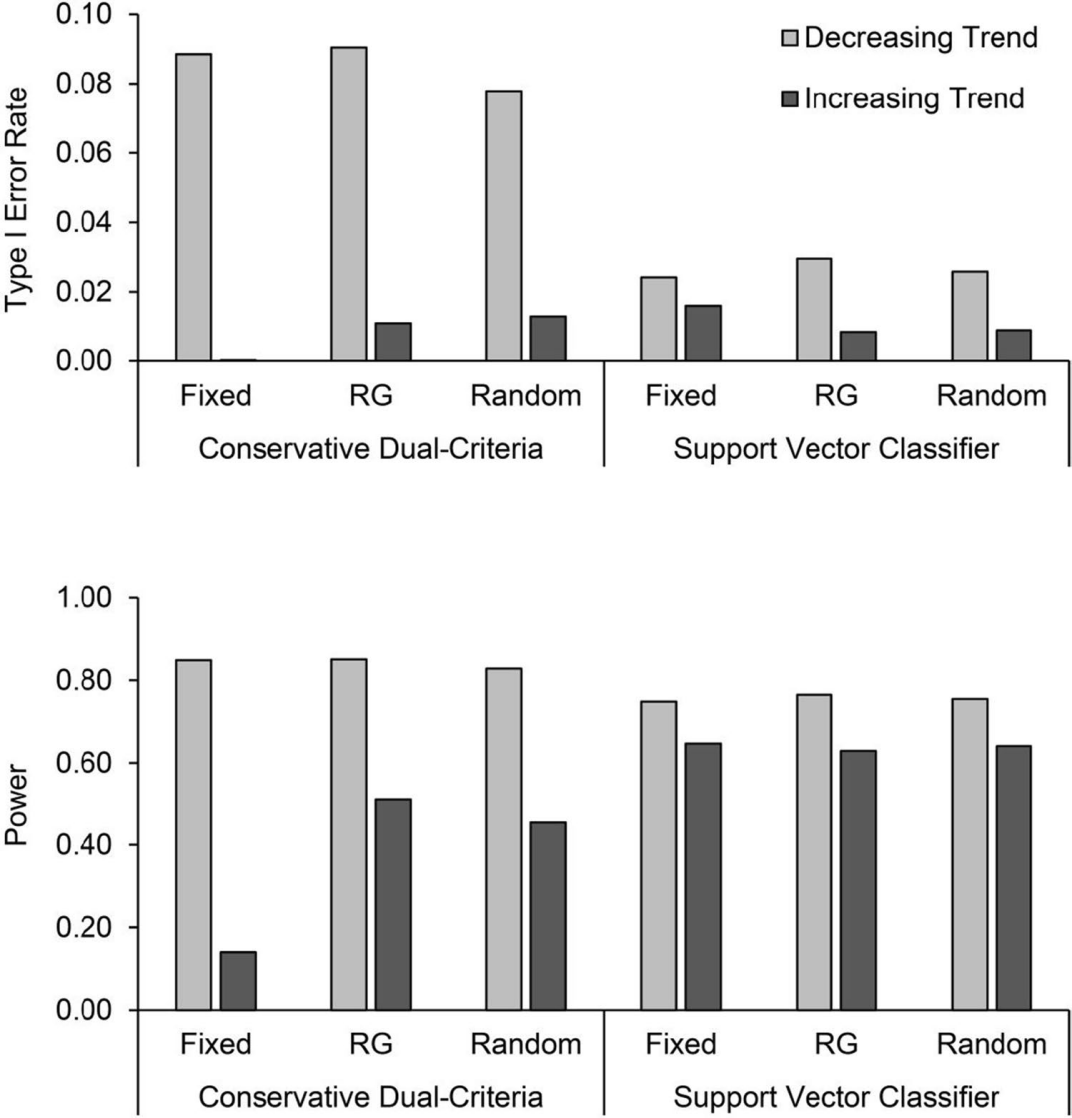
Type I error rate and power across fixed, response-guided (RG), and random baseline lengths when the initial trend was increasing and decreasing

Figure 1 presents the type I error rate and power for the graphs having the minimum number of points (fixed), response-guided graphs, and graphs with a random number of points in phase A. For the CDC method, the responded-guided graphs produced marginally more type I errors than the fixed and random baseline graphs. Graphs with fixed baseline lengths had significantly less power than response-guided and random graphs when applying the CDC. In contrast, the support vector classifier produced consistent results for type I error rate and power regardless of baseline length. Figure 2 depicts type I error rate and power according to the direction of the initial trend. For both methods of analysis, we observed more type I errors and more power when the trend was initially decreasing. Notably, the CDC method produced type I error rates above .05 in this case. The difference between graphs with increasing and decreasing trends was also larger for the CDC method than for the support vector classifier.

Table 2 shows the type I error rate and power based on the minimum number of points in phase A. Generally, increasing the number of points in phase A marginally decreased both type I error rate and power. The only exception is the higher power for fixed graphs with 5 points in phase A using the CDC. Table 3 shows that manipulating the number of points in phase B produced larger differential effects across methods. For the CDC, increasing the number of points in phase B increased type I error rate while keeping a generally consistent power. For the support vector classifier, increasing the number of points in phase B reduced type I error rate (except for the fixed baseline length) while increasing power. In Table 4, type I error increased with autocorrelation for both methods, but it reached above .05 for the CDC only. In comparison, power remained typically consistent for the support vector classifier and was marginally lower for the response-guided and random graphs for the CDC.

**Table 2:** Type I error rate and power across different minimum lengths of phase A for trend stability

	Type I error rate		Power	
	3 points	5 points	3 points	5 points
CDC				
Fixed	.053	.036	.486	.503
Response-guided	.058	.044	.710	.652
Random	.051	.041	.651	.632
SVC				
Fixed	.025	.015	.709	.686
Response-guided	.024	.014	.715	.679
Random	.022	.013	.715	.681

*Note.* CDC: conservative dual-criteria, SVC: support vector classifier.

**Table 3:** Type I error rate and power across different lengths of phase B for trend stability

	Type I error rate		Power	
	5 points	10 points	5 points	10 points
CDC				
Fixed	.030	.059	.498	.492
Response-guided	.038	.064	.671	.690
Random	.033	.058	.634	.649
SVC				
Fixed	.018	.023	.647	.748
Response-guided	.022	.016	.676	.718
Random	.020	.015	.670	.726

*Note.* CDC: conservative dual-criteria, SVC: support vector classifier.

**Table 4:** Type I error rate and power in the absence and presence of autocorrelation for trend stability

	Type I error rate		Power	
	*a* = 0	*a* = 0.4	*a* = 0	*a* = 0.4
CDC				
Fixed	.033	.057	.496	.494
Response-guided	.027	.075	.693	.668
Random	.026	.065	.654	.630
SVC				
Fixed	.011	.030	.696	.699
Response-guided	.007	.031	.701	.694
Random	.007	.028	.699	.697

*Note. a*: autocorrelation values, CDC: conservative dual-criteria, SVC: support vector classifier.

Table 5 examines the effects of manipulating the stringency of the response-guided criterion. When the maximum allowable trend was higher, power and to a lesser extent type I error decreased. This issue was more salient for the graphs analyzed with the CDC. Finally, Table 6 presents the effects of increasing the standardized mean difference. For a SMD of 1, the CDC produced the highest power whereas this pattern was reversed for SMDs of 3 or more (i.e., the support vector classifier performed best). For the CDC, the response-guided graphs produced the highest power closely followed by the graphs with random baseline lengths whereas the fixed graphs produced considerably less power than the two prior categories. For the support vector classifier, power remained similar across fixed, response-guided, and random graphs.

**Table 5:** Type I error rate and power across maximum absolute trend values allowed for trend stability

	Type I error rate		Power	
	15 degrees	30 degrees	15 degrees	30 degrees
CDC				
Fixed	.048	.041	.544	.445
Response-guided	.054	.048	.749	.613
Random	.047	.044	.683	.600
SVC				
Fixed	.024	.016	.716	.679
Response-guided	.021	.017	.709	.685
Random	.019	.016	.709	.687

*Note.* CDC: conservative dual-criteria, SVC: support vector classifier.

**Table 6:** Power across different standardized mean difference values for trend stability

	Standardized mean difference				
	1	2	3	4	5
CDC					
Fixed	0.217	0.427	0.536	0.604	0.689
Response-guided	0.274	0.563	0.757	0.869	0.941
Random	0.259	0.543	0.710	0.812	0.884
SVC					
Fixed	0.176	0.547	0.829	0.948	0.988
Response-guided	0.167	0.545	0.836	0.949	0.989
Random	0.164	0.548	0.836	0.952	0.990

*Note.* CDC: conservative dual-criteria, SVC: support vector classifier.

In general, the results of the first experiment show that waiting for stability with the CDC had minimal impact on type I error, but it considerably increased power when compared to fixed baselines. That said, randomly selecting the number of points in phase A produced similar conclusions to response-guided decisions. Given that the random selection of points may allow for the application of randomization tests (e.g., Levin et al., 2018), randomly selecting the number of points in phase A seems preferable when using the CDC. In contrast, both type I error rate and power remained consistent regardless of baseline length when the support vector classifier analyzed the data. In others word, waiting for stability in trend provided no additional gains for type I error rate and power.

## Experiment 2 – Waiting for stability in variability

Another dimension of single-case graphs that may show stable or unstable patterns involves variability. In single-case designs, “variability refers to the fluctuation of the data (as reflected by the data’s range or standard deviation) around the mean” (Kratochwill et al., 2010, p. 5). Hence, a graph may show no trend, but still remain unstable in terms of variability (Barnard-Brak et al., 2021b). To examine this issue, we replicated our first experiment by creating datasets with high variability and examining the effects of waiting for stability on type I error rate and power.

### Method

The data generation and analysis procedures remained the same as in the first experiment with the following four changes. First, the code generated the points using a random uniform distribution ranging from – 3 to 3, which produced more variable patterns than a normal distribution (i.e., more spread around the mean). This manipulation allowed us to produce graphs with higher variability. Second, the maximum allowable trend value was replaced by a maximum allowable standard deviation value, which was computed using the last three points of phase A. The two cut-off values were standard deviations of 1.0 and 1.5 to reduce the amount of variability. Third, the theoretical standard deviation of the uniform distribution was larger than for the normal distribution (1.73 instead of 1.00). To control for this issue, our code multiplied the SMDs by 1.73 during data generation to allow comparisons across experiments. Finally, we did not repeat the trend analysis (see Fig. 2) as it was not relevant to the type of stability being studied in this experiment.

### Results and discussion

**Fig. 3:**
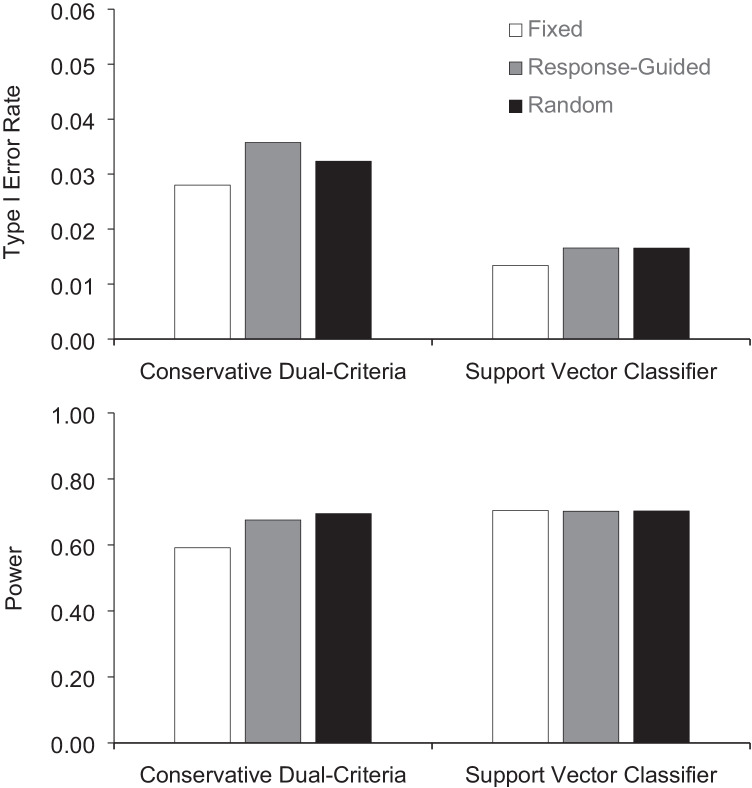
Type I error rate and power across fixed, response-guided, and random baseline lengths for the conservative dual-criteria and support vector classifier for stability in variability

Figure 3 displays the type I error rate (upper panel) and power (lower panel) of waiting for stability in variability during baseline. For the CDC method, having a fixed number of points produced the lowest type I error rate whereas the random number of points produced the highest power. For the support vector classifier, type I error rate and power remained generally consistent regardless of whether we waited for stability to introduce an effect. Tables 7 and 8 present the analysis for the lengths of phases A and B, respectively. Increasing the number of points in phase A marginally decreased type I error rate while increasing power when using the CDC. Manipulating the length of phase A had no systematic effect on the conclusions drawn using support vector classifier. Having a larger number of points in phase B increased both type I error rate and power for the CDC. The support vector classifier with 10 points produced lower type I error rates for the response-guided and random baseline lengths, and higher power for all baseline lengths.

**Table 7:** Type I error rate and power across different minimum lengths of phase A for stability in variability

	Type I error rate		Power	
	3 points	5 points	3 points	5 points
CDC				
Fixed	.029	.027	.512	.671
Response-guided	.040	.031	.645	.706
Random	.035	.030	.669	.720
SVC				
Fixed	.013	.014	.701	.708
Response-guided	.016	.017	.705	.699
Random	.016	.017	.706	.700

*Note.* CDC: conservative dual-criteria, SVC: support vector classifier.

**Table 8:** Type I error rate and power across different lengths of phase B for stability in variability

	Type I error rate		Power	
	5 points	10 points	5 points	10 points
CDC				
Fixed	.021	.035	.590	.593
Response-guided	.027	.045	.667	.684
Random	.025	.040	.683	.707
SVC				
Fixed	.013	.014	.661	.748
Response-guided	.024	.010	.681	.723
Random	.024	.009	.681	.726

*Note.* CDC: conservative dual-criteria, SVC: support vector classifier.

Table 9 presents the effects of autocorrelation while waiting for stability in variability. For the CDC method, autocorrelation was associated with higher type I error rate and lower power. For the support vector classifier, only type I error rate increased with the addition of autocorrelation. Table 10 examines the stringency of our standard deviation stability criterion. The effects of the maximum allowable standard deviation involved a small decrease in type I error rate when the criterion was less stringent criterion (i.e., SD < 1.5) for both methods of analysis. Making the criterion less stringent also decreased power with the CDC method. Finally, Table 11 displays the evolution of power when the value of the SMD increases. Similarly to what was observed during our trend analyses, the CDC had more power than the support vector classifier for low SMDs, but this pattern was reversed for higher SMDs (i.e., 3 or more). Interestingly, the random baseline length showed the most power when applying the CDC method.

**Table 9:** Type I error rate and power in the absence and presence of autocorrelation for stability in variability

	Type I error rate		Power	
	*a* = 0	*a* = 0.4	*a* = 0	*a* = 0.4
CDC				
Fixed	.019	.037	.603	.580
Response-guided	.024	.047	.699	.652
Random	.020	.044	.719	.670
SVC				
Fixed	.005	.022	.708	.701
Response-guided	.010	.023	.707	.697
Random	.010	.023	.706	.700

*Note. a*: autocorrelation values, CDC: conservative dual-criteria, SVC: support vector classifier.

**Table 10:** Type I error rate and power across maximum allowable standard deviations for stability in variability

	Type I error rate		Power	
	SD < 1.0	SD < 1.5	SD < 1.0	SD < 1.5
CDC				
Fixed	.033	.023	.605	.578
Response-guided	.039	.032	.694	.657
Random	.035	.030	.713	.676
SVC				
Fixed	.017	.010	.713	.696
Response-guided	.022	.011	.706	.698
Random	.022	.011	.705	.701

*Note.* CDC: conservative dual-criteria, SVC: support vector classifier.

**Table 11:** Power across different standardized mean difference values for stability in variability

	Standardized mean difference				
	1	2	3	4	5
CDC					
Fixed	0.192	0.509	0.682	0.766	0.808
Response-guided	0.238	0.587	0.770	0.864	0.919
Random	0.234	0.604	0.804	0.892	0.941
SVC					
Fixed	0.146	0.555	0.853	0.971	0.997
Response-guided	0.149	0.553	0.849	0.965	0.995
Random	0.145	0.556	0.852	0.968	0.995

*Note.* CDC: conservative dual-criteria, SVC: support vector classifier. 5266

Once again, the second experiment suggests that the support vector classifier is less affected by changes in baseline stability than the CDC method. The results for the CDC analyses are mixed. Both the response-guided and random baseline lengths produced the highest power, but the response-guided baseline was also associated with the highest type I error rate. Amongst the three baseline lengths, the random length produced the fewest errors overall, which suggest that the benefits of the response-guided decision-making are an artefact of longer baseline phases. For the support vector classifier, the three approaches produced similar patterns of errors and power, which questions the relevance of conducting additional baseline sessions.

## General discussion

The results of our study suggest that the relevance of waiting for baseline stability depends on the method of analysis being applied. For the CDC method, the response-guided and random baseline lengths typically produced fewer errors than the fixed baseline length. In contrast, the three baseline lengths yielded similar results for the support vector classifier. Consistent with prior studies, the support vector classifier produced, on average, fewer decision-making errors than the CDC method (Lanovaz et al., 2020; Lanovaz & Hranchuk, 2021). One potential explanation for the better performance and stability of the support vector classifier is that Lanovaz and Hranchuk (2021) trained their model on graphs with and without trend. Therefore, the support vector classifier “learned” to differentiate between trend and background noise. In comparison, the CDC struggled with power when the trend of data was initially increasing. This observation may be explained by the development of the CDC, which emphasized reductions in type I error rate at the expense of power (Fisher et al., 2003).

For both methods, initially decreasing trends produced higher type I error rates and power. Contrarily, initially increasing trends produced lower type I error rates and power. These results were different from those obtained by Lanovaz and Hranchuk (2021) that observed higher type I error rates in the presence of a trend in the same direction as the expected change. One potential explanation is that trends in Lanovaz and Hranchuk were programmed, which made false positives more likely as the trends remained in the same direction for both phases. Prior studies had shown that increasing the number of points in phase B, but not in phase A, reduced type I error rates (e.g., Falligant et al., 2020; Lanovaz et al., 2017) when using the CDC. Our results were not consistent with prior research: Increasing the number of points in phase A marginally reduced type I error rate while increasing the number of points in phase B increased type I error rate for the CDC in both experiments. For the support vector classifier, increasing the number of points only decreased type I error rate for phase B when we manipulated variability (for two of three baselines). One hypothesis for why the results differ is that prior studies did not systematically introduce trends and variability in their datasets, which may have biased the results. The presence of autocorrelation increased type I error rate for both methods, but to a greater extent for the CDC. Taken together, these results underline the robustness of using machine learning to analyze single-case data with short, fixed baseline lengths.

As discussed previously, waiting for stability did not reduce decision-making errors when using the support vector classifier. Thus, these results suggest that waiting for stability in trend and variability may not be worth the additional time and effort involved in conducting additional baseline sessions. This surprising result may reduce the effort related to baseline data collection as researchers and practitioners may simply set a minimum number of points (e.g., 3 or 5), and then start treatment (or introduce the independent variable) regardless of trend and variability when applying the support vector classifier. Reducing the number of baseline sessions may decrease the costs and limitations of implementing single-case designs in research and practice. For example, researchers and practitioners may want to reduce to a minimum the number of baseline sessions conducted with an individual who exhibits dangerous behavior. Conducting fewer baseline sessions may also allow for the recruitment of more participants in single-case studies by reducing the cost of research per participant. In practice, publicly funded services may be able to reduce wait lists by conducting fewer baseline sessions with each individual. Similarly, individuals on private health plans with a coverage limit may benefit from more treatment sessions if the number of baseline sessions is reduced. As such, reducing the number of baseline sessions could produce beneficial outcomes in both research and practice.

A limitation of our study is that we did not include visual inspection, which is often described as a recommended practice in the analysis of single-case graphs (Lanovaz & Hranchuk, 2021; Manolov & Vannest, 2019). Future research should continue examining how visual inspection is affected by response-guided decision-making. Second, the stability criterion for the second experiment was based on the three last points of phase A. This manipulation was necessary because phase A could have as few as three points. In the future, it may be relevant to examine whether requiring a larger number of points showing stability (e.g., 5) produces differential results. Another limitation is that our pre-set parameters (e.g., autocorrelation, effect size) remained consistent within individual graphs, which introduced some stability. Changing the values of these parameters within graphs appears important to generate more instability and examine the generality of our findings. Finally, we limited our analyses to AB comparisons as it is the basic unit of analysis for many single-case experimental designs. Replicating our study with single-case experimental designs (i.e., reversal designs, multiple baseline designs, and changing-criterion designs) seems essential to examine whether fixed baseline lengths with machine learning would perform the same when the effects are replicated within and across participants. Research on SCDs should also examine the relevance of other recommendations proposed by standards and guidelines to identify their limitations and promote their adoptions by a growing number of researchers and practitioners.

## Footnotes

1 We needed longer time series because the second graph involved achieving stability in baseline. Creating 30-point series ensured that we always had enough points to achieve stability.

## Declarations

### Data and code availability

The data and code generated during the current study are available in the OSF repository, 10.17605/OSF.IO/H7BSG.
